# Ropivacaine suppresses tumor biological characteristics of human hepatocellular carcinoma via inhibiting IGF-1R/PI3K/AKT/mTOR signaling axis

**DOI:** 10.1080/21655979.2021.1995103

**Published:** 2021-11-22

**Authors:** Runze Zhang, Yanhong Lian, Kangjie Xie, Yunfang Cai, Yafei Pan, Yuntian Zhu

**Affiliations:** a Department of Anesthesiology, Cancer Hospital of the University of Chinese Academy of Sciences (Zhejiang Cancer Hospital), Hangzhou, Zhejiang, China; b Department of Anesthesiology, Institute of Basic Medicine and Cancer (IBMC), Chinese Academy of Sciences, Hangzhou, Zhejiang, China

**Keywords:** Ropivacaine, hepatocellular carcinoma, IGF-1R, IGF1R-PI3K-AKT-mTOR signaling, cell cycle, epithelial–mesenchymal transition

## Abstract

Ropivacaine, a common local anesthetic in the clinic, has anti-proliferative and pro-apoptotic effects in numerous cancers, however, the underlying regulatory mechanism of ropivacaine in hepatocellular carcinoma remains unclear. In the current study, human HepG2 cells were stimulated with different ropivacaine concentrations. Cell Counting Kit-8 assay, cell colony formation, and cell cycle were used to monitor cell viability. Cell apoptosis, migration, and invasion were determined by flow cytometry and transwell assays. Tumor xenograft experiments were performed to prove the anti-cancer effect of ropivacaine *in vivo*. A high dose of ropivacaine inhibited proliferation and promoted apoptosis of HepG2 cells in a dose-dependent manner. Ropivacaine challenge also arrested cells in the G2 phase, followed by a decline in the protein expression of cyclin D1 and cyclin-dependent kinase 2, and an increase in p27 levels in HepG2 cells. Additionally, different ropivacaine doses suppressed cell migration and invasion by upregulating E-cadherin expression and downregulating N-cadherin expression. Mechanically, ropivacaine challenge gradually restrained insulin-like growth factor-1 receptor (IGF-1 R) expression and the activities of phosphorylated-PI3K, AKT, and mTOR in HepG2 cells with increased ropivacaine doses. In the tumor xenograft experiment, ropivacaine was confirmed to inhibit tumor growth, accompanied by inhibition of the IGF-1 R/PI3K/AKT/mTOR signaling axis. In conclusion, ropivacaine suppressed tumor biological characteristics and promoted apoptosis, resulting in the suppression of hepatocellular carcinoma progression by targeting the IGF-1 R/PI3K/AKT/mTOR signaling pathway. It is possible that ropivacaine-mediated local anesthesia may be developed as a novel surgical adjuvant drug for treating hepatocellular carcinoma.

## Graphical Abstract

**Figure:**
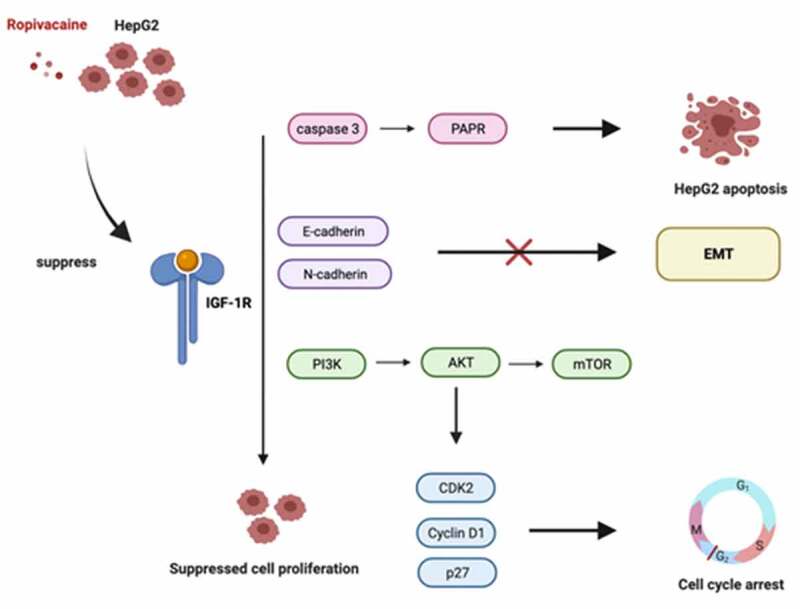

## Induction

It is widely known that local anesthesia is applicable for suitable analgesia drugs and convenient injection [1,2]. Local anesthetic agents are widely used in surgery to reduce or prevent acute pain and cancer pain [3,4]. Several retrospective analyses of patients suggest that local anesthesia application in different surgeries can significantly reduce postoperative pain and improve the cancer survival rate [5,6]. Increasing studies have revealed that local anesthetics inhibit proliferation, invasion, and migration, and promote apoptosis of tumor cells at specific concentrations [7,8]. Therefore, local anesthetic agents combined with surgery may be a breakthrough in cancer therapy.

Ropivacaine, one of the newest amide anesthetic agents, is extensively used in local anesthetic therapy [9,10]. Clinical data have demonstrated that the use of ropivacaine during cancer surgery can reduce cancer recurrence and tumor cell metastasis [11–13]. Additionally, accumulating studies have shown that ropivacaine has anticancer effects in diverse cancers, including hepatocellular carcinoma, and esophageal, colon, and gastric cancers [14–16]. Ropivacaine has been shown to play an effective role in different cancer therapies by inhibiting cell proliferation, migration, invasion, and inducing apoptosis [17,18]. Combination therapy with nutrient deprivation and ropivacaine, which is loaded into tumor-active targeted liposomes, contributes to tumor growth inhibition and cancer pain remission [19]. Local anesthetic ropivacaine may negatively affect tumor reoccurrence and lung tumor metastasis by inducing mitochondrial dysfunction [20]. Nevertheless, the detailed effects of ropivacaine on hepatocellular carcinoma progression and the underlying regulatory mechanisms have not been fully elucidated.

The signaling pathway of insulin-like growth factor 1 (IGF-1)-Phosphatidylinositol-4,5-bisphosphate 3-kinase (PI3K)-protein kinase B (AKT)- mammalian target of rapamycin (mTOR) plays a crucial role in proliferation, differentiation, migration, and metabolism [21]. IGF-1, a member of the tyrosine phosphatase family, has profound effects on antiapoptotic activity [22]. IGF-1 generally binds to IGF-1 receptor (IGF-1 R) to co-regulate cell proliferation and differentiation and activate intracellular kinases, including PI3K [23]. PI3K belongs to the phosphatidylinositol kinase family, which can interact with IGF-1 R to regulate growth, proliferation, migration, motility, and survival through a series of dephosphorylated hydroxyl activities [24]. Activated PI3K can activate the downstream AKT, a serine/threonine-specific protein kinase, which plays a fundamental role in cell survival and apoptosis [25]. MTOR is a serine/threonine secondary phosphorylation kinase involved in cell survival protein synthesis and metabolism [26]. It has been reported that local anesthetic lidocaine or mepivacaine can impair human dermal fibroblast proliferation of elders by inhibiting IGF-1 R, which is the curial regulator of wound healing [27]. Studies have shown that the anesthetic agent bupivacaine induces apoptosis via the reactive oxygen species-mediated PI3K/Akt pathway [28], and lidocaine can block the signal transduction of PI3K/Akt to exert neuroprotective effects [29]. Levobupivacaine can inhibit breast cancer cell proliferation and lead to apoptosis by inhibiting the PI3K/Akt/mTOR signaling pathway [30]. In summary, the IGF-1 R-PI3K-Akt-mTOR signaling pathway is over-activated in numerous cancers and is involved in the local anesthetic agent-mediated anti-proliferative and pro-apoptotic progression.

Thus, we hypothesized that the local anesthetic ropivacaine may be implicated in hepatocellular carcinoma development by regulating the IGF-1 R-PI3K-Akt-mTOR signaling pathway. In the present study, we aimed to investigate the effects of ropivacaine on tumor biological behaviors in hepatocellular carcinoma using cell and animal models. Furthermore, changes in the IGF-1 R/PI3K/AKT/mTOR axis were assessed in ropivacaine-challenged cells and tumor xenograft animals. This study reveals that the local anesthetic ropivacaine may be involved in impairing tumor biological characteristics of human hepatocellular carcinoma.

## Materials and methods

### Cell culture and treatment

HepG2 cells were cultured in modified Eagle’s medium (MEM, PM150410, Procell Life Science & Technology, Wuhan, China) supplemented with 10% fetal bovine serum (FBS, 164,210–500, Procell Life Science & Technology) and maintained in an incubator at 37°C with 5% CO_2_. The medium was updated every two days. Cells were induced with 0.1, 0.5, 1, 2, 5, 10, 3.769, 7.538, and 15.076 mM ropivacaine hydrochloride monohydrate (106,828, ChemeGen, Shanghai, China) according to the manufacturer’s protocol [31,32]. The cells were then harvested for the subsequent experiments.

### Cell viability assay

Cell viability was evaluated using the Cell Counting Kit-8 (CCK-8) assay (HY-K0301, MedChemExpress, NJ, USA). Briefly, HepG2 cells were seeded at a density of 2,500 cells/well in 96-well plates and treated with different ropivacaine concentrations (0.1, 0.5, 1, 2, 5, and 10 mM) for 48 h [33]. Subsequently, 10 μl CCK-8 solution was added to each well and maintained in an incubator for an additional 4 h at 37°C. Absorbance values were obtained at a wavelength of 450 nm using a spectrophotometer (UV-1800PC, Shanghai Mapada Instruments, Shanghai, China).

### Cell cycle and analysis

A cell cycle detection kit (KGA512, KeyGen Biotech, Nanjing, China) was used to evaluate the cell cycle. In brief, cells were digested with 0.1% trypsin (15,050,065, Gibco, TX, USA) and fixed in 700 μl 80% cold ethanol at 4°C overnight. The next day, cells were treated with 100 ul RNase at 37°C for 30 min and stained with 400ul PI (50 ug/ml) for 30 min at 4°C in the dark in accordance with standard procedures [34]. The cell cycle was analyzed by flow cytometry, and Flow Jo software was used to measure the proportions of cells in different phases.

### Cell apoptosis assay

Cell apoptosis analysis was conducted by flow cytometry using the Annexin V-EGF/PI Cell Apoptosis Detection Kit (KGA1026, KeyGen Biotech). In brief, treated cells were washed twice with phosphate-buffered saline, resuspended in Annexin V-FITC/PI cocktail, and incubated with the cocktail for 15 min at 37°C in the dark [35]. The apoptotic cell rate was determined by flow cytometry.

### Migration and invasion assay

The transwell assay was used to assess cell migration and invasion. The transwell chamber was pre-treated with or without Matrigel (356,234, BD Biosciences, CA, USA), and 5 × 106 cells were cultured in the upper chamber with FBS-free modified Eagle’s medium (MEM) [35]. Simultaneously, the bottom chamber was added to the medium contaaining 10% FBS. Cells were cultured for 24 h and stained with 0.2% crystal violet. Migration and invasion abilities were observed using a microscope (ECLIPSE Ts2, Nikon, Tokyo, Japan).

### Colony-forming assay

HepG2 cells were seeded in a 6-well plate at 1000 cells/well for 24 h. The following day, the cells were incubated with different concentrations (3.769, 7.538, and 15.076 mM) of ropivacaine for 2 weeks. Subsequently, the colonies were stained with 0.5% crystal violet for 20 min, and the colonies were photographed and counted [36].

### Western blot

Total protein was extracted using Radio Immunoprecipitation Assay (RIPA) buffer (P0013B, Beyotime, Jiangsu, China) containing 1 × protease inhibitor cocktail [36]. Total protein was separated by 12% SDS-PAGE and transferred onto polyvinylidene fluoride membranes (IPFL00010, Merck, MA, USA). The bands were incubated overnight with primary antibodies at 4°C. Primary antibodies are listed below: cleaved caspase 3 (AF7022, Affinity, CA, USA; 1:1000), caspase 3 (AF6311, Affinity; 1:1000), cleaved poly ADP-ribose polymerase (PARP; AF7023, Affinity; 1:1000), PARP (ab191217, Abcam, Cambridge, UK; 1:1000), cyclin D1 (AF0931, Affinity; 1:1000), cyclin dependant kinase 2 (CDK2; AF6237, Affinity; 1:1000), p27 (AF6324, Affinity; 1:1000), epithelial €-cadherin (AF0131, Affinity; 1:1000), neural (N)-cadherin (AF4039, Affinity; 1:1000), IGF1R (AF6125, Affinity; 1:1000), p-PI3K (AF3241, Affinity; 1:1000), p-AKT (ab38449, Abcam; 1:1000), p-mTOR (AF3308, Affinity; 1:1000), and GAPDH (ab9485, Abcam; 1:1000). The following day, the bands were incubated with goat anti-rabbit IgG (H + L) HRP (S0001, Affinity; 1:5000) for 1 h at room temperature. The results were visualized with ECL reagent (WBKlS0010, Merck), photographed with Gel Imager System, and analyzed using Image J software (1.8.0, National Institutes of Health, USA).

### Xenograft experiments in nude mice

Male BALB/C nude mice (N = 10, 8-weeks old) were purchased from Changzhou Cavens Laboratory Animal Co., Ltd. (Changzhou, China). All mice were randomly divided into two groups: a control group and a ropivacaine-exposed group. All animal experiments were approved by the Ethics Committee of Zhejiang Cancer Hospital, and according to standard institutional guidelines. Nude mice were subcutaneously injected with HepG2 cells (4 × 10^6^) into the right side of the armpits. After tumors grew up to a volume of 50 mm^3^, one group was administered ropivacaine (20 mg/kg/day) and the control mice were injected with 0.9% normal saline. Tumor volume was measured every 3 days, and all mice were euthanized 24 h after the final administration on day 25 [37]. Finally, the tumor diameters and weights were calculated to generate growth curves.

### Statistical analysis

All experiments were repeated at least three times. All data are shown as the mean ± SD and were analyzed using GraphPad Prism 9 software (CA, USA). Statistical differences were determined by Student’s t-test between two groups and by one-way analysis of variance among diverse groups. Statistical significance was set at P < 0.05.

## Results

Accumulating evidence suggests that ropivacaine possesses anti-proliferative and pro-apoptotic effects in several tumors; however, its regulatory role in hepatocellular carcinoma remains unclear. Thus, this study aimed to investigate the role of the local anesthetic ropivacaine on hepatocellular carcinoma progression. Combined with previous studies, we assumed that the local anesthetic ropivacaine potentially participated in hepatocellular carcinoma progression by regulating the IGF-1 R-PI3K-Akt-mTOR signaling pathway. In this study, the effects of ropivacaine on tumor biological behaviors in hepatocellular carcinoma were confirmed using cell and animal models. Furthermore, changes in the IGF-1 R/PI3K/AKT/mTOR axis were assessed in ropivacaine-challenged cells and tumor xenograft animals. Our findings demonstrate the anti-tumor effects of ropivacaine in hepatocellular carcinoma. The highlights of this research are as follows:

## Ropivacaine suppresses cell proliferation and promotes cell apoptosis in Human HepG2 Cells

**Figure 1.**
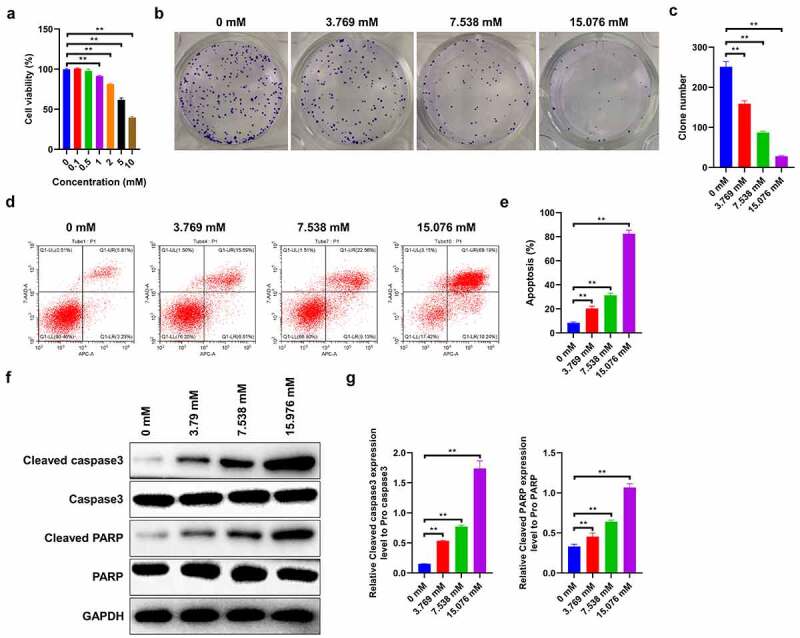
Effects of ropivacaine on cell proliferation and apoptosis of human HepG2 cells. (a) HepG2 cells incubated with different concentrations of ropivacaine (0.1 mM, 0.5 mM, 1 mM, 2 mM, 5 mM, 10 mM) for 48 h. Cell viability as determined by Cell Counting Kit-8 assay kit, **P < 0.01. (b)-(c) Cell growth was valued by the colony formation assay with different concentrations of ropivacaine (3.769 mM, 7.538 mM and 15.076 mM) treatment (B) and quantitative analysis as presented in the right (C), **P < 0.01. (d)-(e) After treating with 3.769 mM, 7.538 mM and 15.076 mM ropivacaine, cell apoptosis was conducted by flow cytometry using the Annexin V-EGF/PI cell apoptosis detection kit (D) and quantitative analysis as presented in the right (E), **P < 0.01. (f)-(g) Apoptosis-related proteins such as cleaved caspase 3 and cleaved PARP as detected by Western blotting (F) and quantitative analysis as presented in the right (G), **P < 0.01

In order to explore the effects of ropivacaine on cell viability, HepG2 cells were treated with different doses (0, 0.1, 0.5, 1, 2, 5, and 10 mM) for 48 h. The CCK-8 assay reveals that, beyond 1 mM, ropivacaine markedly inhibits cell viability, while lower ropivacaine concentrations display humble effects on HepG2 cell proliferation (IC50 = 7.538 mM, Figure 1a). Therefore, 3.769, 7.538, and 15.076 mM gradient ropivacaine concentrations, as described in the literature [31] were chosen for the following cellular experiments. Subsequently, the colony forming assays indicated that 3.769 mM ropivacaine notably weakens the clone number and survival ability of HepG2 cells, and the inhibitory effect increases with the increase in ropivacaine dose, showing the strongest inhibition in 15.076 mM ropivacaine-exposed cells (Figure 1b, p < 0.01). Furthermore, flow cytometry results show that the apoptotic proportion of HepG2 cells increases with increasing ropivacaine concentration in a dose-dependent manner (Figure 1c-1d, p < 0.01). Apoptosis-related proteins, including cleaved caspase 3 and cleaved PARP, are elevated in ropivacaine-challenged cells, and the effect was dose-dependent (figure 1f-1g, p < 0.01). Taken together, the above results indicate that ropivacaine can repress proliferation and promote apoptosis in HepG2 cells in a dose-dependent manner.

## Ropivacaine arrests cells in G2 phase

**Figure 2.**
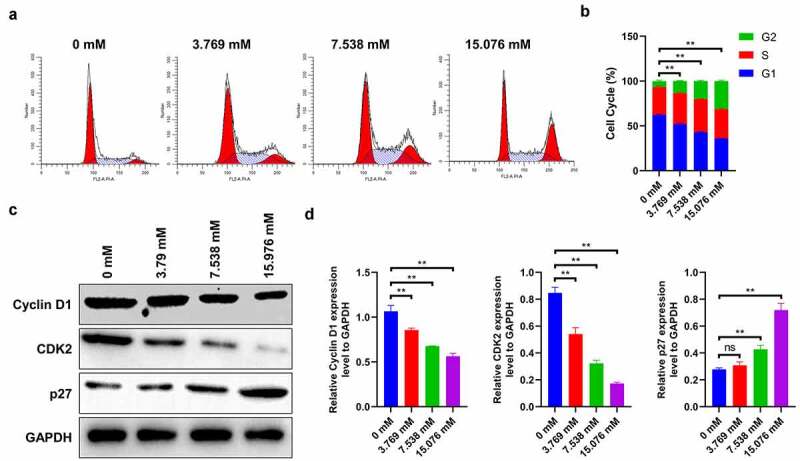
Role of ropivacaine on cell cycle of HepG2 cells. HepG2 cells were firstly exposed to 3.769 mM, 7.538 mM or 15.076 mM ropivacaine for 48 h. (a)-(b) Cell cycle was analyzed by cell cycle detection kit using flow cytometry (A) and quantitative analysis as presented in the right (B), **P < 0.01. (c)-(d) Cell cycle-related protein levels of Cyclin D1, CDK2 and p27 as monitored by Western blotting (C) and quantitative analysis as presented in the right panel **(D)**, **P < 0.01; ns, no significant

Next, HepG2 cells were exposed to different ropivacaine doses (3.769, 7.538, and 15.076 mM) for 24 h to monitor its role in cell cycle regulation. It is seen that 3.769 mM ropivacaine significantly reduces the proportion of G1-phase cells and induces the accumulation in G2 phase, a phenomenon that is most conspicuous in 15.076 mM-treated cells (Figures 2a-b), suggesting that ropivacaine induces cell arrest in the G2 phase (P < 0.01). Correspondingly, the immunoblotting assay reveals that the protein expressions of cyclin D1 and CDK2 (as markers of G1 phase) sharply decrease, and p27 (as the G2 phase marker) gradually decreases with the increase in ropivacaine concentration (Figure 2c-2d, p < 0.01). It is possible that ropivacaine mediates the anti-proliferative effect by inducing G2 cell cycle arrest and cell apoptosis.

## Ropivacaine inhibits migration and invasion of HepG2 cells

**Figure 3.**
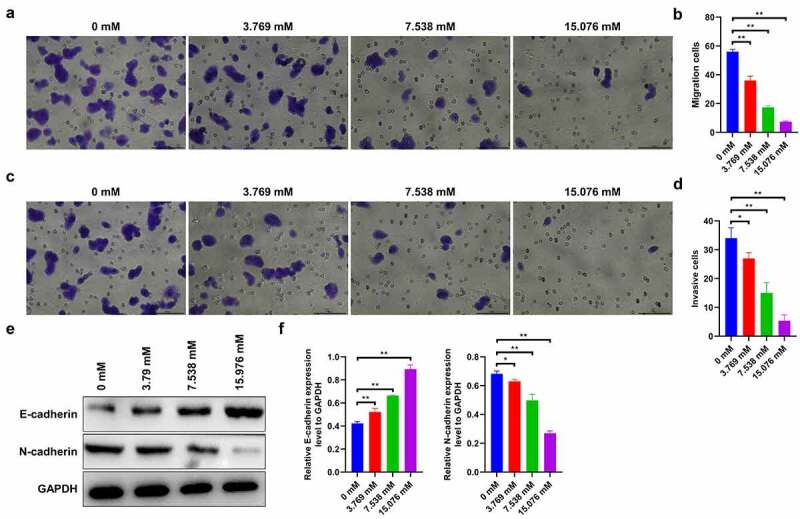
Migration and invasion capabilities in ropivacaine-exposed HepG2 cells. (a)-(b) HepG2 cells were treated with different concentrations of ropivacaine (3.769 mM, 7.538 mM and 15.076 mM), then the Transwell migration assay was employed to detect the migration ability (A) and quantitative analysis as presented in panel (B), **P < 0.01. Scale bar: 100 μm. (c)-(d) Transwell invasion assay was utilized to evaluate the invasion ability (C) and quantitative analysis as presented in panel (D), **P < 0.01; *P < 0.05. Scale bar: 100 μm. (e)-(f) The protein expression levels of E-cadherin and N-cadherin as detected by Western blotting (E) and grayscale analysis was accounted in panel (F), **P < 0.01; *P < 0.05

Furthermore, we investigated the effects of ropivacaine on cell migration and invasion. The results show that 3.769 mM ropivacaine significantly reduces the number of migrated cells compared to the control group, showing the strongest effect against cell migration at high doses (15.076 mM) of ropivacaine-challenged cells (Figure 3a-3b, p < 0.01). Consistently, 3.769 mM ropivacaine evidently attenuates cell invasion capability (P < 0.05), and this inhibitory effect is dose-dependent (Figure 3c-3d, p < 0.01). Moreover, western blotting was used to assess the levels of epithelial–mesenchymal transition (EMT)-related proteins, including E-cadherin and N-cadherin, which were considered as the ‘switch’ of tumor migration and invasion [38]. The results show that E-cadherin is discernibly upregulated (P < 0.01), while N-cadherin is downregulated with different doses of ropivacaine exposure in HepG2 cells (Figure 3e-3f, p < 0.05). Collectively, these data imply that ropivacaine suppresses the migration and invasion capabilities of HepG2 cells.

## IGF1R-PI3K-AKT-mTOR signaling in ropivacaine-treated HepG2 cells

**Figure 4.**
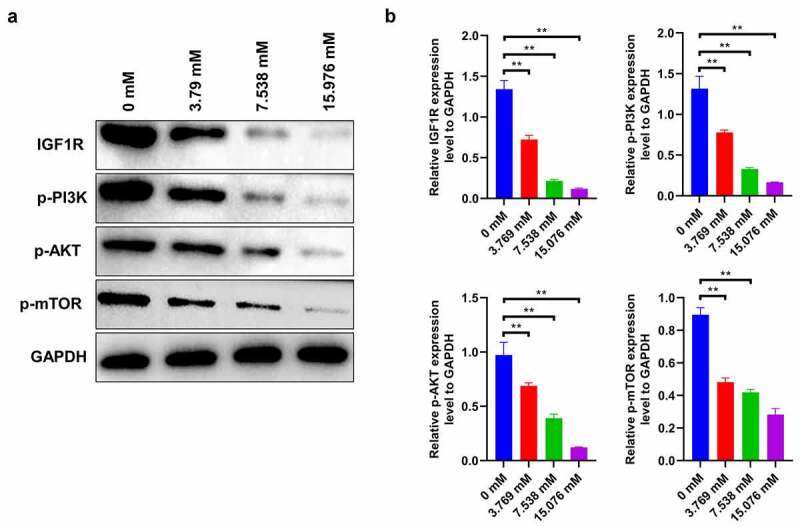
Changes of IGF1R-PI3K-AKT-mTOR signaling in HepG2 cells treated with ropivacaine. HepG2 cells were firstly exposed to 3.769 mM, 7.538 mM or 15.076 mM ropivacaine for 48 h. (a)-(b) Protein expression levels of IGF-1 R, p-PI3K, p-AKT, and p-mTOR detected by Western blotting (A) and grayscale analysis was accounted in panel (B), **P < 0.01

Generally, activation of the IGF1-PI3K-AKT-mTOR signaling pathway facilitates the proliferation and migration of cancer cells [21]. In this study, ropivacaine-exposed HepG2 cells show a significant decrease in IGF-1 R expression in a dose-dependent manner, followed by a gradient descent in the activities of downstream molecules, including phosphorylated PI3K, AKT, and mTOR (Figure 4a-4b, p < 0.01). This finding indicates that ropivacaine may restrain the biological characteristics of liver tumor cells by targeting the IGF1R-PI3K-AKT-mTOR signaling axis.

## Ropivacaine represses tumor growth *in vivo*

**Figure 5.**
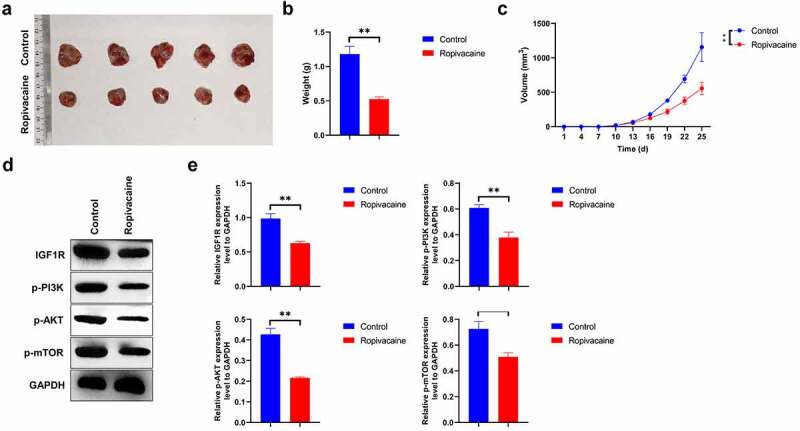
Therapeutic efficacy of ropivacaine on tumor growth of tumor xenografts. (a) HepG2 cells were implanted into BALB/c nude mice, and the overall appearances of the tumor tissues of control group and ropivacaine-challenged group were photographed. (b) The measurement of individual tumor weight in each group, **P < 0.01. (c) The tumor length (l) and width (w) were recorded every three days and tumor volumes were computed as (L x W x W)/2, **P < 0.01. (d)-(e) Protein levels of IGF-1 R, p-PI3K, p-AKT, and p-mTOR as detected by Western blotting (D) and quantitative analysis as presented in panel (E), **P < 0.01

To determine whether ropivacaine had an anti-tumor effect *in vivo*, HepG2 cells were injected subcutaneously into male BALB/C nude mice to establish a tumor xenograft model. The mice were euthanized when HepG2 cells were successfully implanted for 25 days. Ropivacaine challenge effectively inhibits subcutaneous tumor growth in total volume and weight compared to the negative groups (Figure 5a-5c, p < 0.01). Subsequently, we isolated tumor proteins and detected the expression of IGF-1 R-PI3K-AKT-mTOR signaling molecules and found that ropivacaine treatment notably reduces the level of IGF-1 R **and** inactivates the PI3K-AKT-mTOR signaling transduction (Figure 5d-5e, p < 0.01). All the above data indicate that ropivacaine suppresses tumor growth in a xenograft model *in vivo*, potentially by regulating the IGF1R-PI3K-AKT-mTOR signaling axis.

## Discussion

Our study demonstrated that ropivacaine was capable of inhibiting cell viability and enhancing apoptosis in HepG2 cells, and the potential anticancer mechanism of ropivacaine in hepatocellular carcinoma may involve the IGF-1 R/PI3K/AKT/mTOR axis. Abundant evidence indicates that local anesthetics may be beneficial for anti-cancer [39,40]. Our findings may provide a new therapeutic approach for anti-hepatocellular carcinoma progression.

Rapid proliferation and protection from apoptosis are typical cancer cell characteristics [41,42]. Ropivacaine is related to cancer progression by inhibiting cell proliferation and inducing cell apoptosis in breast cancer, glioma, and lung cancer [43–45]. Moreover, ropivacaine has been shown to exert vital functions, such as the induction of apoptosis, in hepatocellular carcinoma [46]. In the present study, we found that ropivacaine suppressed the viability and growth of HepG2 cells in a dose-dependent manner. Consistently, the effective anti-proliferative doses of ropivacaine were similar to those reported previously [32,47]. The reduction of G1/G0 phase cells is prevalent in the majority of cancer cells, which eventually display phenotypes of S phase arrest or G2/M phase arrest [48,49]. In our study, flow cytometry showed that ropivacaine treatment arrested cells in the G2 phase and decreased the proportion of G1 phase cells. Consistent with our findings, ropivacaine has also been shown to reduce the G1 phase population in cervical, breast, colon, and pancreatic cancers [46,50–52]. Collectively, our results demonstrate that ropivacaine decreases cell viability, likely by arresting the cell cycle in the G2 phase.

Furthermore, ropivacaine significantly promotes apoptosis in multiple cancers [33,44,53]. PARP and caspase 3 are considered crucial components of the apoptotic cascade [54,55]. In the current study, ropivacaine challenge induced cell apoptosis by elevating the expression of cleaved caspase 3 and cleaved PARP in a dose-dependent manner. Similarly, ropivacaine has been found to activate the caspase 3 signaling pathway in hepatocellular carcinoma [33], and ropivacaine can activate the cleaved PARP and decrease Bcl-2 protein to induce apoptosis in other cancer cells [56,57]. Therefore, ropivacaine-restrained cell viability may be involved in drug-induced programmed apoptosis.

E-cadherin and N-cadherin are calcium-dependent transmembrane glycoproteins that mediate cell adhesion [38]. It is increasingly acknowledged that E-cadherin and N-cadherin are closely related to the progression of invasion and metastasis [38]. Accumulating evidence suggests that ropivacaine inhibits the migration and invasion of diverse cancers, such as esophageal and papillary thyroid cancers [16,36] and ultimately achieves the goal of anti-tumor therapy. Ropivacaine treatment upregulates E-cadherin expression and represses vimentin expression in glioma cells, resulting in metastasis inhibition [44]. The anti-metastatic effect of ropivacaine may be via impeding Src-activation and intercellular adhesion molecule-1 phosphorylation, thus hindering EMT progression [58]. Correspondingly, our findings showed that ropivacaine effectively inhibited the migration and invasion of HepG2 cells, along with a decrease in E-cadherin and an increase in N-cadherin. E-cadherin conventionally acts as an invasion suppressor in the majority of tumors and is generally downregulated; in contrast, N-cadherin, regarded as the promoter of oncogenes, is upregulated in metastatic cancer cells; these two cadherins maintain the balance of invasion [59]. Overall, our results further confirm that ropivacaine can inhibit cell metastasis in hepatocellular carcinoma, possibly by regulating EMT progression.

The IGF-1/PI3K/AKT/mTOR signaling pathway may be one of the important mechanisms of cancer [60]. There have been several reports that local anesthetics regulate the PI3K/AKT/mTOR pathway to mediate cancer cell activities, including apoptosis and autophagy [30,61]. Notably, ropivacaine was found to suppress cell growth and survival in chronic myeloid leukemia by reducing the PI3K/AKT/mTOR pathway [62], and ropivacaine stimulates apoptosis of hepatocellular carcinoma by mediating Akt activity and caspase 3 cleavage [62]. In this study, along with the reduction of cell viability *in vitro* and tumor growth *in vivo*, the signaling transduction of IGF-1 R/p-PI3K/p-AKT/p-mTOR was abolished in ropivacaine-treated HepG2 cells and ropivacaine-challenged tumor-bearing mice *in vivo*. Synthetically, ropivacaine may regulate cell proliferation and apoptosis through the IGF-1 R/PI3K/AKT/mTOR axis to exert anti-cancer effects.

However, numerous issues require further clarification. First, the activators of the IGF-1 R/PI3K/AKT/mTOR signaling axis, such as the IGF-1 R agonist, PI3K/AKT signaling activator, or mTOR activator, should be employed to determine the association between ropivacaine and the IGF-1 R/PI3K/AKT/mTOR pathway in liver tumor cells. Moreover, gene data array analysis of clinical samples should be performed to further judge the conclusion. Further clinical trials are required to investigate whether ropivacaine can be used as a combined adjuvant drug for surgical resection of hepatocellular carcinoma. Thus, we would further discover the detailed regulatory mechanism in ropivacaine-mediated anti-tumor effects by combining signal activators or more clinical verification.

## Conclusion

Ropivacaine was shown to inhibit tumor biological characteristics while inducing apoptosis in hepatocellular carcinoma. In fact, the anti-tumor functions are mediated by ropivacaine, potentially via inactivation of the IGF-1 R/PI3K/AKT/mTOR signaling pathway. This research provides an innovative proposal for applying local anesthetics to limit liver cancer progression. However, more potent clinical data are needed to confirm these findings. It is possible that local anesthetic agents may be used as a combination drug therapy to damage the function of tumor biological characteristics of human hepatocellular carcinoma.

## Data Availability

The datasets generated and/or analyzed during the current study are not publicly available due to the need of permission from the IRB but are available from the corresponding author on reasonable request zhangrz@zjcc.org.cn

## Abbreviation

IGF-1, Insulin-like growth factor 1; PI3K, Phosphatidylinositol-4,5-bisphosphate 3-kinase; AKT/PKB, Protein kinase B; mTOR, mammalian target of rapamycin; CDK2, Cyclin-dependent kinase 2; ROS, reactive oxygen species; EMT, epithelial–mesenchymaltransition; PARP, poly ADP-ribose polymerase; RIPA, Radioimmunoprecipitation Assay; MEM,modified eagle medium; FBS, Fetal Bovine Serum; CCK-8, Cell Counting Kit-8.
